# The mitochondrial genome of *Xanthochlorus tibetensis* (Diptera: Dolichopodidae)

**DOI:** 10.1080/23802359.2021.1872444

**Published:** 2021-02-12

**Authors:** Juan Wang, Yutong Ji, Lisheng Zhang, Mengqing Wang

**Affiliations:** Institute of Plant Protection, Chinese Academy of Agricultural Sciences, Beijing, China

**Keywords:** Mitochondrial genome, Dolichopodidae, Xanthochlorinae, phylogenetics

## Abstract

The long-legged fly *Xanthochlorus tibetensis* belongs to the subfamily Xanthochlorinae of Dolichopodidae. The mitogenome of *X. tibetensis* was sequenced, it is the first representative complete mitogenome from this subfamily. This mitogenome is 15,580 bp in size, includes 13 protein-coding genes, 22 transfer RNAs, and two ribosomal RNAs. All genes have the same location and coding strand as in other published species of Dolichopodidae. Nucleotide composition is biased toward A and T, which together made up 77.6% of the entire genome. Bayesian inference strongly supported the monophyly of Empidoidea, Empididae and Dolichopodidae, with the phylogenetic relationships within Empidoidea: ((Dolichopodinae + Xanthochlorinae) + Neurigoninae) + ((Trichopezinae + (Empidinae + Oreogetoninae)) + Ocydromiinae).

## Introduction

*Xanthochlorus* is the only genus in subfamily Xanthochlorinae Aldrich, which is one predatory group. This genus is a very rare group in the Dolichopodidae, distributed in the Holarctic and Oriental regions with 16 known species. *Xanthochlorus tibetensis* was described in 2015 with material from subtropical forest in Tibet (China) (Xi et al. 2015), and was first found from Tajikistan when we checked materials from Tajikistan this summer. This special species is mainly yellow in thorax and abdomen with yellow bristles, bearing nearly quadrate first flagellomere. Male abdominal tergite 6 is large and genitalia is rather large and mostly exposed with modified cercus, and female abdomen is wide and obtuse apically (Yang et al. 2006, 2011; Xi et al. 2015).

The adult specimens of *X. tibetensis* used for this study were collected in Sari Nay Village of the Nurobad region in Tajikistan in 2013 by Jun Chen & Jian Yao and identified by Mengqing Wang. Specimens were deposited in the Natural Enemy Insects Museum (Accession Number: NI2013-12) of the Institute of Plant Protection, Chinese Academy of Agricultural Sciences (IPPCAAS) (Room 311, Plant Protection Building). Total genomic DNA was extracted from a whole body (except head) specimen using the QIAamp DNA Blood Mini Kit (Qiagen, Germany) and stored at −20 °C until needed. 1 μg of genomic DNA was used to generate libraries with an average insert size of 350 bp, which were sequenced using the Illumina NovaSeq 6000 platform (Berry Genomics, Beijing, China) with 150 bp paired-end reads on one sample per flow-cell lane. A total of 24,593,710 raw paired reads were generated. The quality of all sequences was checked using FastQC (http://www.bioinformatics.babraham.ac.uk/projects/fastqc). Clean reads were assembled using the MitoZ v2.4 pipeline (Meng et al. 2019). The assembled genome was annotated using the MITOS webserver with the invertebrate genetic code (Bernt et al. 2013).

The complete mitogenome of *X. tibetensis* is 15,580 bp (GenBank accession number: MT949691) and encodes 13 PCGs, 22 tRNA genes, and 2 rRNA genes. All genes have the same location and coding strand as in other published species of Dolichopodidae (Hou et al. 2019; Qilemoge et al. 2020). Nucleotide composition is biased toward A and T, with 77.6% A + T content (A = 39.4%, T = 38.2%, C = 12.8%, G = 9.6%). The A + T content of PCGs, tRNAs, and rRNAs is 76.0, 77.2, and 80.3%, respectively. All PCGs (12 of 13) initiate with ATN codons (6 with ATG, 4 with ATT, 1 with ATA, and 1 with ATC) while *NAD1* starts with GTG. The typical termination codons TAA and TAG are respectively assigned to ten and two PCGs. However, *NAD4* terminates with TA as an incomplete stop codon which is common in Diptera species (Kang et al. 2016; Li et al. 2016).

**Figure 1.**
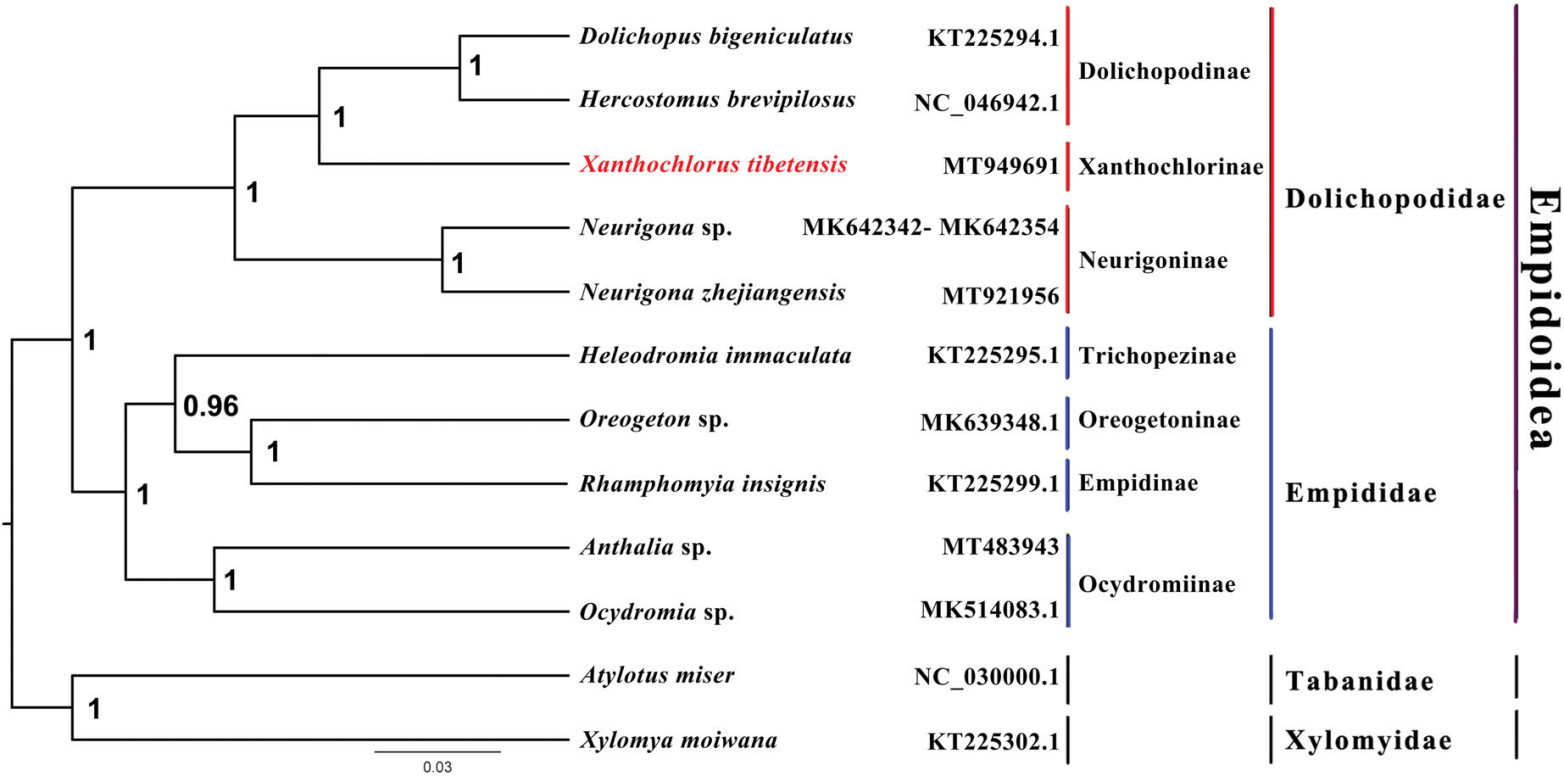
Bayesian phylogenetic tree of 12 Diptera species. The posterior probabilities are labeled at each node. GenBank accession numbers of all sequences used in the phylogenetic tree have been included in figure and corresponding to the names of all species.

Phylogenetic analysis was performed based on the nucleotide sequences of 13 PCGs from 12 Diptera species. Sequences were aligned using MAFFT v7.313 (Katoh and Standley 2013) and the Bayesian Inference (BI) tree was constructed with MrBayes 3.2.6 (Ronquist et al. 2012), which was run for 2,000,000 generations and sampled from every 100 generations. The CAT + GTR model selected by ModelFinder (Kalyaanamoorthy et al. 2017) was applied. Bayesian posterior probabilities were calculated after discarding the first 25% of the trees. The phylogenetic result strongly supported the monophyly of Empidoidea, Dolichopodidae and Empididae. Monophyletic Dolichopodinae, Xanthochlorinae and Neurigoninae were grouped as a monophyletic Dolichopodidae, which was sister group to a monophyletic Empididae that consists of Empidinae, Trichopezinae, Oreogetoninae and Ocydromiinae in this study (Figure 1). Monophyly of Empididae and Dolichopodidae is consistent with previous phylogenetic results (Wang et al. 2016). This is the first sequenced mitogenome from the genus *Xanthochlorus*, which is a very rare group in Dolichopodidae. The mitogenomic data of *X. tibetensis* could provide the important information for the further studies of Dolichopodidae phylogeny. We hope to find more species in East and West Asia regions, and get more chance to observe their predation on small pests. Further studies are needed to sequence more species from the subfamily Xanthochlorinae, as well as other subfamilies, which will enhance our understanding of molecular phylogeny in Dolichopodidae.

## Data Availability

The genome sequence data that support the findings of this study are openly available in GenBank of NCBI at (https://www.ncbi.nlm.nih.gov/) under the accession no. MT949691. The associated BioProject, SRA, and Bio-Sample numbers are PRJNA681987, SRP295457, and SAMN16976141, respectively.
